# Extended Reality Telemedicine Collaboration System Using Patient Avatar Based on 3D Body Pose Estimation

**DOI:** 10.3390/s24010027

**Published:** 2023-12-20

**Authors:** Matko Šarić, Mladen Russo, Luka Kraljević, Davor Meter

**Affiliations:** Faculty of Electrical Engineering, Mechanical Engineering and Naval Architecture, University of Split, Ruđera Boškovića 32, 21000 Split, Croatia; mrusso@fesb.hr (M.R.); lkraljev@fesb.hr (L.K.); davor.meter.00@fesb.hr (D.M.)

**Keywords:** extended reality, telemedicine system, avatar, 3D body pose estimation

## Abstract

Recent advances in extended reality (XR) technology have opened the possibility of significantly improving telemedicine systems. This is primarily achieved by transferring 3D information about patient state, which is utilized to create more immersive experiences on VR/AR headsets. In this paper, we propose an XR-based telemedicine collaboration system in which the patient is represented as a 3D avatar in an XR space shared by local and remote clinicians. The proposed system consists of an AR client application running on Microsoft HoloLens 2 used by a local clinician, a VR client application running on the HTC vive Pro used by a remote clinician, and a backend part running on the server. The patient is captured by a camera on the AR side, and the 3D body pose estimation is performed on frames from this camera stream to form a 3D patient avatar. Additionally, the AR and VR sides can interact with the patient avatar via virtual hands, and annotations can be performed on a 3D model. The main contribution of our work is the use of 3D body pose estimation for the creation of a 3D patient avatar. In this way, 3D body reconstruction using depth cameras is avoided, which reduces system complexity and hardware and network resources. Another contribution is the novel architecture of the proposed system, where audio and video streaming are realized using WebRTC protocol. The performance evaluation showed that the proposed system ensures high frame rates for both AR and VR client applications, while the processing latency remains at an acceptable level.

## 1. Introduction

Telemedicine systems are a well-known technology aimed at improving healthcare and reducing costs. The need for telemedicine was recognized during the COVID-19 pandemic, when physical interaction with patients was risky. Until recently, telemedicine relied on 2D modalities, that is, video conferencing, which substituted cell phone consultation. This includes audio and single-viewpoint video transmission. The drawback of the 2D approach is the limited availability of information about the patient’s condition in comparison with a physical examination. With the recent advances in augmented reality (AR), virtual reality (VR), and mixed reality (MR), where all these technologies can be encompassed by the term extended reality (XR), it is feasible to transmit more information about patient states, primarily by including the third space dimension (3D). VR and AR technologies have practical advantages and disadvantages. While VR provides a highly immersive experience that is particularly suitable for educational purposes, users’ field of view is blocked. On the other hand, AR adds computer-generated information to the user’s field of view, but it lacks interaction with 3D objects. MR, and consequently XR solutions, overlay 3D objects onto a semi-transparent screen, preserving the user’s view of the physical environment and making these technologies good candidates for telemedicine collaboration. A 3D human avatar can be a valuable tool in telemedicine because it allows healthcare providers to remotely interact with patients in a more immersive and personalized way. Annotations of 3D models (human avatars) in extended reality (XR) can provide a valuable tool for telemedicine. In XR, healthcare professionals can use virtual annotations to highlight specific areas of a patient’s anatomy or medical imaging, which can be viewed and manipulated in real-time. For example, a radiologist can use annotations to mark abnormalities in a CT scan or MRI, which can be viewed by other specialists at different locations. This approach can help with collaborative diagnosis and treatment planning and provide a more detailed understanding of the patient’s condition. Additionally, annotations in XR can be used for patient education, allowing healthcare professionals to explain medical conditions and procedures to patients using a 3D avatar or model. This approach can be particularly useful for patients who may have difficulty understanding medical terminology or who have limited access to traditional education materials. Overall, annotations of 3D models in XR can enhance the telemedicine experience, providing healthcare professionals with a more detailed understanding of a patient’s condition, facilitating collaboration and communication, and improving patient outcomes. Holographic communication is an emerging technology that extends traditional video conferencing with real-time 3D representations of participants. The typical steps in holographic communications include real-time 3D capture of an object or person, data processing for 3D model creation, its transmission through a network, presentation of the transmitted model on the receiving end, and real-time interaction with the 3D model. Although holographic communication has the potential to improve remote interaction in medicine and healthcare applications, it faces significant implementation challenges [1]. Three-dimensional data capture should be performed in real time; however, consequently, the precision of the acquired data is lower. For this purpose, time-of-flight (ToF) cameras or stereo cameras are used, but because of their narrow field of view, a multi-camera setup is often required. Three-dimensional reconstruction is performed on a large amount of the captured data, which implies longer processing time and, consequently, greater latency. The transmission of 3D data is related to three main challenges: ultra-high bandwidth, ultra-low latency, and network optimization. The use of 5G networks can only partially satisfy the transmission speed requirements of holographic communication systems. End-to-end transmission latency is influenced by all steps, from local site data capture to remote site rendering. Currently, holographic communication systems generate a latency of several hundred milliseconds [1], which is greater than the recommended value of 50–100 ms [2]. In this paper, we propose an XR-based telemedicine collaboration system that enables local doctors to consult with remote specialists where patients are represented as 3D human body avatars in a shared XR space. The local doctor, using the AR application running on an AR headset (Microsoft HoloLens 2), starts the remote session with a remote specialist who works with VR applications running on a VR headset (HTC Vive Pro). First, Web Real-Time Communication (WebRTC) is established between the AR side and VR side, where a remote specialist sees a real-time camera stream (captured by a HoloLens 2 camera or webcamera) of the patient. This stream is processed on the backend part of the system to obtain the data necessary for patient 3D avatar control. The data are subsequently sent to the AR and VR sides so that they can see the same 3D avatar matching the patient pose. In this way, both sides are present in the shared XR space, allowing interaction with the patient avatar in the form of virtual hands. For this purpose, hand pose data are exchanged between the AR and VR sides in real time. Since the 3D avatar of the patient is transmitted to the remote site, the proposed approach is an example of a holographic communication system. The main contribution of this paper is the use of 3D body pose estimation for the creation of a patient avatar. This approach is different from the approaches described in related work, where the patient is captured with depth camera(s) and reconstructed as a 3D model. In the proposed approach, 3D patient reconstruction is avoided, which eliminates the need for specialized equipment and reduces computational and network resources. In this way, we reduce processing time and the amount of data needed for 3D avatar creation, which are typical challenges met in holographic communication systems. Our system represents a simpler and more robust solution in comparison with other approaches from the literature. Another contribution is the novel system architecture exploiting the WebRTC protocol for audio and video streaming. This ensures lower latency, which is especially critical in video streaming applications. Additionally, in this way, direct peer-to-peer communication is established between the AR side and VR side, eliminating the need for additional servers. The paper is organized as follows. In Section 2, an overview of the related work is given. Section 3 provides a detailed description of the proposed system, and Section 4 presents the results obtained by system testing. Conclusions are given in Section 5.

## 2. Related Work

Regarding the usage of XR technology in telemedicine applications, we can mention the solution proposed in [3], where an MR teleconsultation system was realized. Azure Kinect DK is exploited on the patient side, while a doctor uses Microsoft HoloLens 2, which allows manipulation of 3D organ models and medical images. In this way, doctors can explain symptoms and educate patients through real-time video communication realized through Microsoft Teams. In [4], a platform for real-time remote medical consultations that combines VR and AR technologies was introduced. On the patient side, an RGB+D video is captured with a Microsoft Kinect device and sent, together with vital patient information, to the remote location where streamed data are visualized to the expert using a stereoscopic display. Augmented feedback is presented on the patient side using a projector previously calibrated with Kinect. Carbone et al. [5] developed an AR-based telemedicine platform using a see-through head mounted display (HMD) on the remote or local clinician side. In this way, both HMDs show the same image in which the hands of a remote specialist are overlaid on the actual scene to guide the local clinician. In [6], the authors described a 3D teleconsultation system intended for preclinical emergency scenarios. A depth camera captures the patient environment, and from this information, 3D reconstruction is performed on the remote expert side. Guidelines are transmitted to the patient side using a shared avatar representation of the expert, which is presented to the local clinician via an AR HMD. The system was compared to the 2D video teleconsultation approach on the task of electrocardiogram electrode placement, and it was shown that higher accuracy was obtained using the proposed 3D approach. In [7], an MR system for surgical telementoring was introduced. A remote expert utilizes a VR operating room, where gestures and annotations on a 3D patient model are sent to remote novice surgeons using the AR interface. The novice side is equipped with depth cameras capturing the operating room, with one attached to a surgical lamp to scan the patient’s body. The novice surgeon uses the Microsoft HoloLens 2 device to run the AR interface, which has only passive elements to avoid distractions. The remote expert is equipped with a VR headset, HTC Vive Pro, and IMU-equipped gloves. Roth et al. [8] presented a mixed reality teleconsultation system intended for usage in intensive care units. It consists of three modules: a reconstruction module, a local expert module, and a remote expert module. The reconstruction module creates a local point cloud of the intensive care unit using frames obtained from six RGB-D cameras. The remote expert module uses a VR HMD to allow experts to join the reconstructed intensive care unit space. Remote experts are represented with avatars in the local expert module where the AR system is used. In [9], a mixed reality (MR) system for surgical telementoring was proposed. The patient is captured with a Kinect depth camera and reconstructed in a remote virtual environment where an expert surgeon can interact with the 3D patient model. The local surgeon is guided by 3D annotations projected in AR as well as the gestures of the avatar representing the expert surgeon that are shown using AR. Kalbas et al. [10] introduced an AR-based surgical telementoring system. Microsoft HoloLens 2 is used to share the operating surgeon’s field of view, and accurate 3D annotations are provided with satisfactory accuracy. The application of a telemedicine system for remote patient monitoring is described in [11], where the use of a teleguided portable ultrasound with ultrasound image analysis was proposed for COVID-19 patients. Using the proposed approach, individuals with limited medical backgrounds can achieve high accuracy in detecting COVID-19. Hill [12] demonstrated the use of the Microsoft HoloLens 2 device to improve outcomes in patients receiving negative pressure wound therapy. The bedside nurse used a HoloLens 2 to remotely consult wound care personnel, who could load previous wound images into the local nurse’s field of view and perform 3D drawings to guide the procedure. The study group using AR technology had fewer complications than the control group. Borresen et al. [13] proposed an augmented reality telerehabilitation system for the remote examination of upper extremity strength and range of motion. The solution is based on two Kinect depth cameras that capture 3D videos of the patient’s body and a Force Dimension Omega.3 Haptic Controller device for transmission of the patient’s force. The results show that remote assessment performed remotely with the proposed system has promising agreement with in-person diagnoses.

## 3. Proposed Method

**Figure 1:**
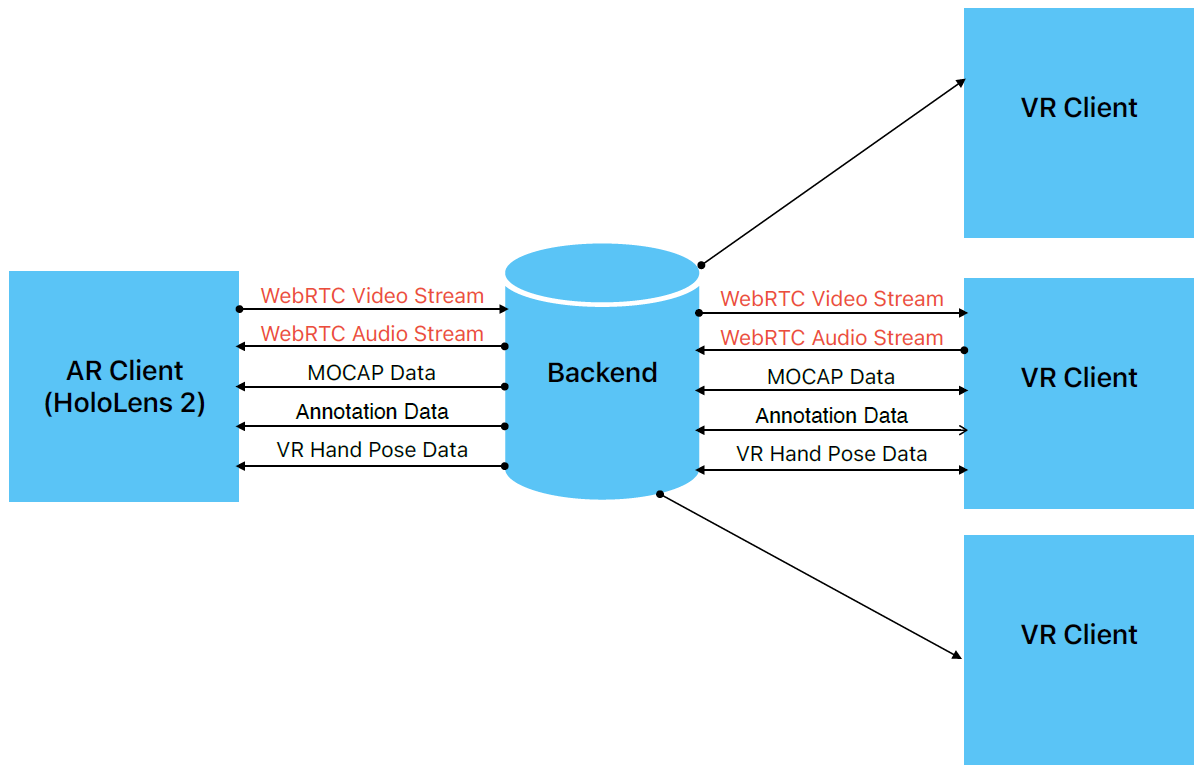
Architecture of the proposed system.

The purpose of the proposed solution is to enable patient examination by remote experts without the need for physical presence. The system consists of three components (Figure 1): a user application with an AR interface (AR client), used by a local doctor performing a physical examination of a patient; a user application with a VR interface (VR client), used by a remote expert; and an XR collaboration system (backend) running on a workstation/server. A local doctor using AR glasses (Microsoft HoloLens 2) starts a telemedicine session to receive guidelines from the remote expert. The webcamera view of the patient is streamed to the remote location where the expert uses VR glasses (HTC VIVE Pro). The camera stream is also processed on the server to obtain the data needed for controlling the 3D avatar of the patient. These data are subsequently sent to the VR and AR sides to match the pose of the avatar with the patient’s pose. During collaboration, both sides share the common XR space and observe the same avatar. Additionally, in real time, the AR and VR sides exchange data related to the relative position of user/control modalities in the AR/VR interface. In this way, the transmission of the hands/controllers is enabled, and both users see virtual hands as a form of interaction with a 3D avatar. Both sides are able to annotate the 3D model. Microsoft HoloLens 2 is an augmented reality (AR) headset developed for mixed reality applications where the focus is on blending virtual elements with the real world. It is equipped with various sensors, including an RGB camera, a depth camera, head tracking cameras, eye-tracking cameras, and an IMU unit. It uses see-through waveguide lenses with 2048 × 1080 resolution and supports hand-tracking with various gesture readings. One feature that is exploited in the proposed system is holographic remoting, where the app interface is seen on a HoloLens 2 device, but it is actually running on a PC to utilize more powerful hardware to avoid a decrease in the frame rate. The HTC Vive is a VR headset featuring a dual AMOLED display with a resolution of 1080 × 1200 per eye. The refresh rate is 90 Hz with a 110-degree field of view. One of the important features of such systems is the use of a room-scale tracking system based on the base stations, which track the headset and handheld controllers used for interaction and navigation in the VR space.

### 3.1. Avatar Control

**Figure 2:**
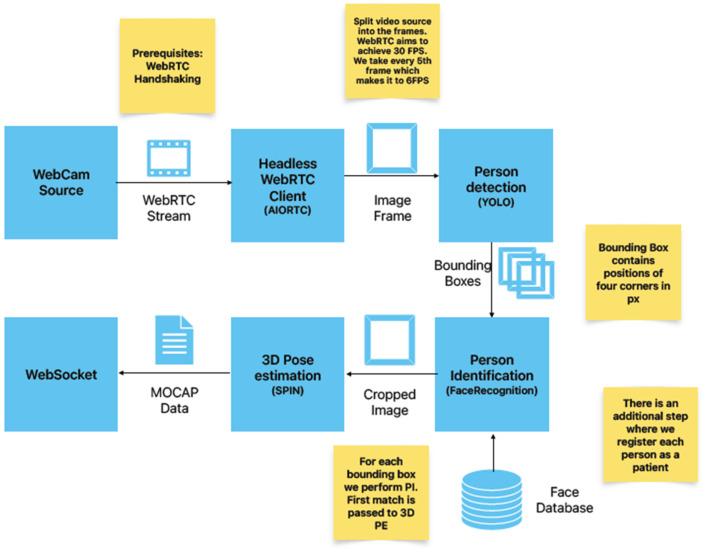
Avatar control workflow.

By creating a 3D avatar of a patient, doctors can better visualize and understand the patient’s physical condition, which can help with diagnosis and treatment planning. Additionally, patients can use 3D avatars to convey information about their symptoms and medical history to healthcare providers, even if they are not physically present in the same location. A human/patient avatar can help doctors better understand a patient’s physical condition by providing a more detailed and visual representation of the patient’s anatomy. By creating a 3D avatar of the patient, doctors can examine the avatar from different angles, zoom in on specific areas, and manipulate the avatar to better understand the patient’s condition. This approach can be particularly useful when physical examination is limited, such as in telemedicine, or when the condition is complex and difficult to visualize. The avatar control workflow, shown in Figure 2, is an essential part of the proposed XR collaboration system. Several microservices are needed to establish real-time communication and process the camera stream.

The REST service is employed for user authentication. The real-time communication (RTC) service used is based on the WebRTC protocol. WebRTC was chosen for this purpose because it offers several advantages:

- Low latency, which is especially important for real-time interaction in videoconferencing and telemedicine applications
- Peer-to-peer (P2P) communication, where video streams are sent directly between users, which eliminates the need for a server.
- High-quality supporting adaptive bitrates needed to handle varying network conditions.
- Open source and standardized: the developers’ community ensures constant improvements.

In the proposed system, the RTC service is implemented as a headless WebRTC client that has two functions: the first one is audio and video communication between the AR and VR sides and the processing of the camera stream, and the second is the usage of different computer vision algorithms on the server side. The headless WebRTC client unpacks the video stream into individual frames, and every computer vision algorithm is represented as an individual microservice. The message exchange service, based on the WebSocket protocol, transfers the following JSON messages between the AR and VR sides:

- The WebRTC data (OFFER, ANSWER, ICECANDIDATES) needed for establishing a RTC connection.
- MOCAP data obtained by pose estimation, which are used for avatar control in the XR space (shared 3D space).
- Collaborative cross-platform hand pose data are 3D vectors that enable the visualization of the user’s hands/controllers. In this way, the AR and VR sides are able to virtually collaborate on the patient’s 3D model.
- Annotation data: the remote or local expert can annotate a point on an avatar and send its position and textual description.

The person detection service is realized using a real-time object detection algorithm from the MediaPipe library [14]. This object detector supports several models to balance the processing time and the detection accuracy. The recommended model is EfficientDet-Lite0 from the EfficientDet model family, which is introduced in [15]. It utilizes a weighted bi-directional feature pyramid network (BiFPN) for feature fusion and compound scaling of the resolution, depth and width for all backbone, feature network, and box/class prediction networks. The headless WebRTC client unpacks frames from the camera stream that are sent to the object detector, giving the person bounding box as output. The patient identification service exploits the Python library’s face recognition [16], ensuring that the pose estimation data and annotation data are combined with the correct person representing the patient. This is especially important in cases where a connection is re-established or when there are several people present at the scene.

### 3.2. Pose Estimation

Three-dimensional pose estimation is the most important and most challenging step in the proposed system. Generally, pose estimation can be realized in several different ways. Two-dimensional pose estimation involves estimating the position of body joints in a two-dimensional image. It can be performed using methods such as single-person pose estimation or multi-person pose estimation. Three-dimensional pose estimation determines the position of body joints in three-dimensional space. It is usually realized using a combination of 2D image information and depth information. Kinematic pose estimation gives the full-articulated pose of a person by considering the kinematic relationships between body parts. Human pose estimation has many practical applications, such as in human–computer interactions, sports analysis, and medical diagnosis. It can also be used for tracking the movements of people in surveillance systems or for creating realistic virtual avatars. In the proposed system, a 3D pose estimation service is realized using the model proposed in [17]. This method involves the use of a 3D pose estimation system for the body, face, and hands using only monocular images. It outputs in the form of the SMPL-X model [18], which combines the skinned multi-person linear (SMPL) model [19], which represents the human body as a collection of interconnected joints, and the linear blend skinning model with the face and hands model. In this way, a low-dimensional representation of the human body, hands, and face is obtained. The SMPL-X model is able to model shape variations and body deformations with low-dimensional shape and pose parameters. The SMPL-X model extends the SMPL model to include articulated hands and an expressive face. The SMPL-X model, denoted by *W*, can be expressed as follows:

(1) $$V_{w}=W\left(\phi_{w},\theta_{w},\beta_{w},\psi_{f}\right)$$

where $\phi_{w}\in R^{3}$ is whole body global orientation, $\theta_{w}\in R^{(21+15+15)\times 3}$ refers to whole body pose parameters, $\beta \in R^{10}$ represents shape parameters, and $\psi_{f}\in R^{10}$ are facial expression parameters. Pose parameters θ are divided into 21 body parameters, 15 parameters for the left hand, and 15 parameters for the right hand. Pose parameters θ are defined with the angle-axis notation that defines relative rotation to the parent joint. The SMPL-X model has the mesh structure $V\in R^{10435\times 3}$. Three-dimensional body joint locations could be calculated using the regression function *R* on $V_{w}$:

(2) $$J_{w}^{3D}=R_{w}\left(V_{w}\right)$$

where $J_{w}^{3D}\in R^{(22+15+15)\times 3}$. The hand model is defined using only hand parts from the SMPL-X model:

(3) $$V_{h}=W\left(\phi_{h},\theta_{h},\beta_{h}\right)$$

where $\theta_{h}\in R^{3\times 15}$ represents hand pose parameters, $\beta_{h}$ are hand shape parameters, and $\phi_{h}$ is the global orientation of the hand mesh. The output of the hand model is the hand mesh structure $V_{h}^{778\times 3}$, containing hand vertices extracted from the SMPL-X hand area. Three-dimensional hand joints can be calculated by regression:

(4) $$J_{h}^{3D}=R_{h}\left(V_{h}\right)$$

where $J_{h}^{3D}\in R^{21\times 3}$ includes the wrist, 5 fingertips, and 15 finger joints. The 3D hand pose estimation model is built as an end-to-end deep neural network architecture, and is defined as follows:

(5) $$\left[\phi_{h},\theta_{h},\beta_{h},c_{h}\right]=M_{H}\left(I_{H}\right)$$

where $I_{h}$ stands for the input RGB image cropped to show only the hand region and $c_{h}\left(t_{h},s_{h}\right)$ represents the set of weak perspective camera parameters used for the projection of the obtained 3D model onto the input image. The 3D body pose estimation model is given by:

(6) $$\left[\phi_{b},\theta_{b},\beta_{b},c_{b}\right]=M_{B}\left(I_{b}\right)$$

where $I_{b}$ represents the image cropped around the person’s body. For this model, the method presented in [20] is exploited. Firstly, 2D joint localization is performed on input using a pretrained model, such as OpenPose. After that, the model utilizes a deep neural network to perform regression of the SMPL-X parameters. The regressed parameter values are further refined via an iterative optimization routine, where the model is aligned with the image based on 2D keypoints. This step minimizes the mismatch between the projected 3D joints in the SMPL-X model and the 2D joint positions in the image. The 3D face-estimation model presented in [21] is used to obtain the face poses $\theta_{f}$ and facial expressions $\psi_{f}$:

(7) $$\left[\theta_{f},\psi_{f}\right]=M_{F}\left(I_{f}\right)$$

The final output of the described model is formed by combining outputs from the face, hands, and body modules into the SMPL-X representation.

The output of the model contains the positions of the body joints, and these data are used for kinematic graph calculations according to the SMPL-X model, where each joint movement is expressed as a function of the other joints’ movements. A kinematic graph gives rotation data for 24 joints that represent MOCAP data, which are sent using a message exchange service to the AR and VR sides and mapped to the avatar. The Z-anatomy 3D model is exploited to visualize different systems of the human body (cardiovascular, skeletal, nervous, etc.). This model is modified in Blender to obtain the skin mesh, which is connected with the skeletal system of the SMPL-X. Regarding the accuracy of the 3D pose estimation model [17] exploited in our system, an evaluation by the authors shows that the mean vertex-to-vertex distance (V2V) in millimeters is 63.5 mm, where the vertex refers to the SMPL-X model.

## 4. Results

**Figure 3:**
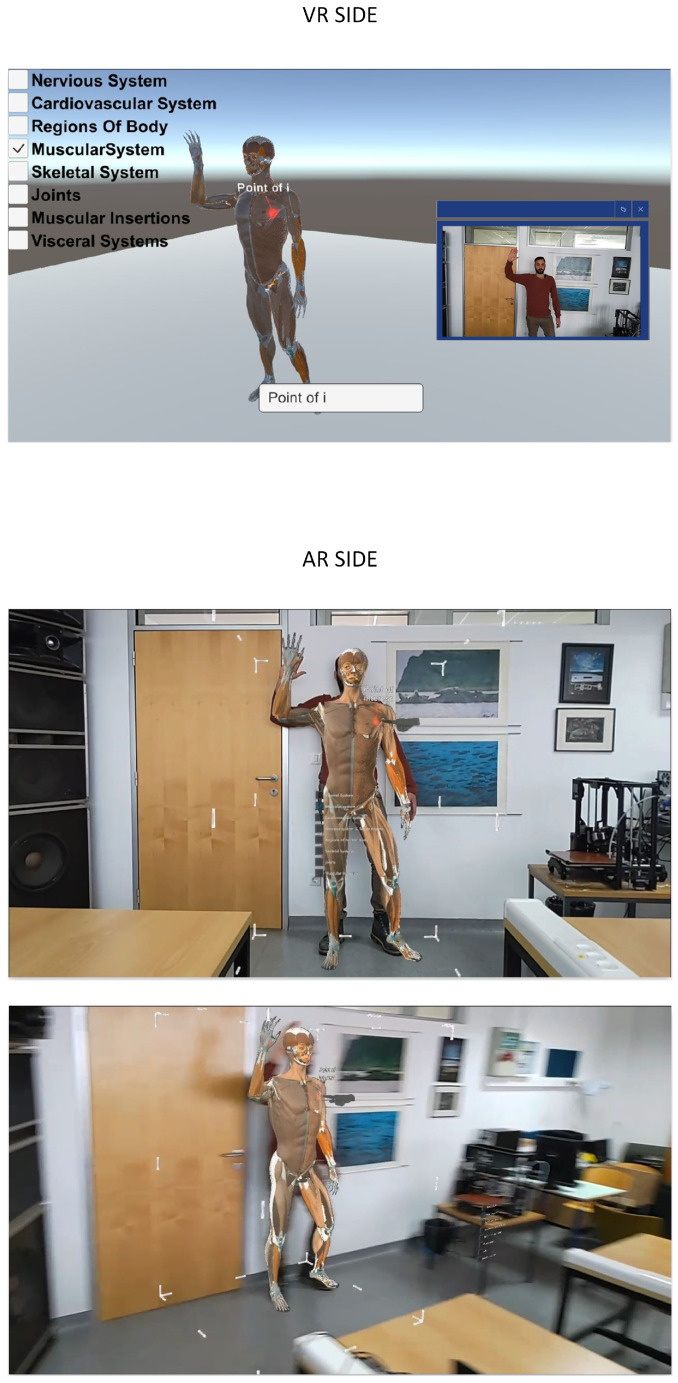
VR and AR client views. The red point on the avatar represents point of interest, and the virtual hand (in gray color) represents the remote specialist hand.

**Figure 4:**
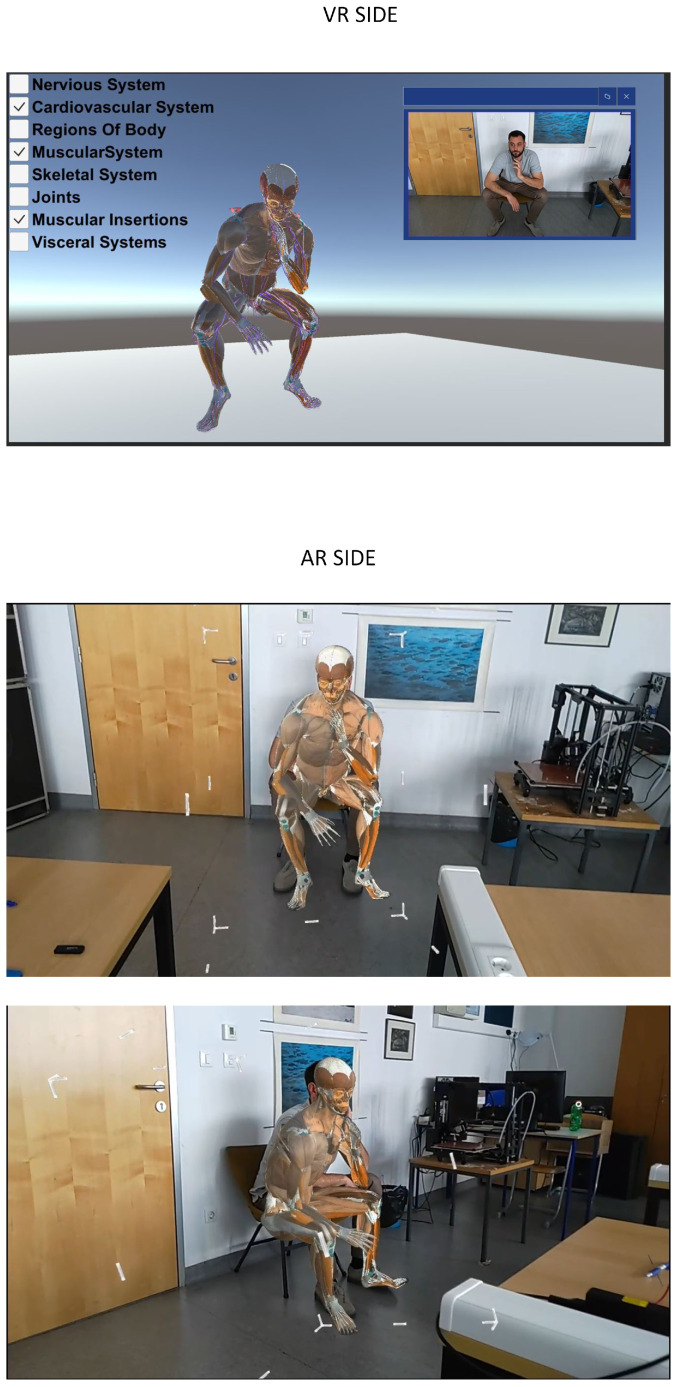
VR and AR client view for patient in a sitting pose.

To better illustrate the usage of the proposed system, examples of the AR client view and VR client view are shown in Figure 3 and Figure 4, respectively. The VR client shows the 3D patient avatar presented to the remote specialist wearing the HTC VIVE Pro VR headset. This view also includes WebRTC preview of the camera from the AR side. The AR client view, presented to the local doctor using Microsoft HoloLens 2, overlays the 3D patient avatar with the physical environment view. The AR client enables avatar translation, rotation, and scaling to better match the patient’s body in a real environment. Figure 3 shows an example of annotation on the 3D avatar as well as a virtual hand that guides the local doctor. Figure 4 illustrates that the system is robust to varying patient poses, with the avatar matching the patient in the sitting position. Since 3D body pose estimation is used instead of 3D reconstruction, the matching accuracy is not sufficient for tasks requiring high precision, such as surgical procedures. Interaction with a patient avatar is achieved with a virtual hand, which represents the hand of a remote specialist. Different human body systems can be chosen for visualization according to the doctor’s preferences.

Since the time delay of avatar movement is critical for system usability, data streaming experiments were performed to determine the times needed for each step in the avatar control workflow. The execution times for different services are shown in Table 1. Frame grabbing, person detection, person identification, and 3D pose estimation services were run on the following server configurations: AMD CPU Ryzen 9 5900X, RAM 3200 Mhz 4 × 16 GB, GPU NVIDIA RTX A5000 24 GB, and SAMSUNG SSD 980 PRO 1TB M.2. Peer-to-peer delay (WebRTC) and MOCAP data message exchange delay are measured on the LAN network. The unity 3D model transformation on the VR side was run on a laptop with the following configurations: CPU Intel(R) Core(TM) i5-9300H CPU @ 2.40GHz, GPU NVIDIA GeForce GTX 1650, SSD NVMe Micron_2200 _MTFD _16GB, and RAM DDR4 16 GB 3.2 GHz. The VIVE Pro headset was connected to a laptop to present the 3D model to a remote specialist. HoloLens 2, used on the AR side, can run applications using onboard hardware or a PC in holographic remoting mode. It can be seen that running the client application on the laptop, which is the only available option on the VR side, is significantly faster compared to the running time when the client application runs on HoloLens 2 hardware. It should be noted that, in the holographic remoting mode, the HoloLens camera is not accessible from the code, and therefore an external webcamera is used for patient capture. As expected, WebRTC communication significantly contributes to the overall delay, which has values ranging from 340 to 560 ms. The individual computer vision service requires time ranging from 15 to 60 ms. Compared to approaches using 3D reconstruction for avatar creation, the usage of 3D body pose estimation in the proposed system reduces the amount of transferred data and requires less processing time, which is comparable to the time needed for other computer vision algorithms in avatar control workflow. Although direct comparison of latency with other systems from the literature is not possible due to the specific features of each architecture, the obtained values are comparable to the latency of other holographic communication systems, which have values of several hundred milliseconds [1]. The mixed reality telemedicine system proposed in [8] has similar latency values (300–400 ms). It uses depth cameras and 3D reconstruction of the intensive care unit, but the reconstruction module, network transmission, and streaming processing are implemented in C++/CUDA to reduce processing time. In the proposed approach, Python implementations of computer vision services for avatar control are used to determine the possibility of further reducing the time delay by C++ implementation of some tasks.

The frame rate obtained in client applications on the VR and AR sides is a critical factor for system usability; therefore, the frame rate (Table 2) is measured in two lightning scenarios (light and no light) and the human body system (Z-anatomy model) chosen for visualization. A constant frame rate is obtained for the AR client application (60 fps) running on a laptop (AR-remote) and for the VR client application (120 fps) also running on the same laptop (VR-build). It can be seen that, for the AR client running on the HoloLens 2 hardware, the frame rate is variable and depends on the type of body system chosen for visualization. The main difference between the proposed method and the existing approaches is the construction of the patient avatar using 3D body pose estimation from 2D images. A similar system was proposed in [8]; however, instead of a patient, remote experts are represented as avatars in the 3D-reconstructed intensive care unit. The body poses of remote clinicians are obtained with trackers attached to the ankles, wrists, and hip, while in the proposed approach, additional sensors are not needed to construct a patient avatar. In [7], a 3D reconstruction of the patient body is performed using depth images captured by a Microsoft Azure Kinect camera. Since the Kinect camera is attached to a surgical lamp, this setup is suitable for patients in a lying position. The proposed solution does not require a depth camera and could also be used for other patient poses (Figure 4). Limitations of the proposed system include the accuracy of 3D body pose estimations, which affect the alignment of the 3D avatar with the patient’s body. Because of that, the proposed system is not suitable for surgical applications, but it could be exploited for remote patient evaluation, such as remote triage, nonsurgical remote medical interventions, and educational purposes.

## 5. Conclusions

In this paper, we introduce an XR-based telemedicine collaboration system based on the 3D avatar modality for patient body representation. Instead of relying on 3D reconstructions of the patient’s body using depth cameras, 3D body pose estimation from 2D images is utilized for this purpose. In this way, there is no need for an array of depth cameras that are preinstalled and calibrated in a controlled environment, which reduces the cost and system complexity. Additionally, the processing time and amount of transferred data are reduced compared to those of approaches utilizing depth cameras and 3D reconstruction. The remote expert (VR side) and local doctor (AR side) exchange information by interacting with the same patient avatar in a shared XR space. This process is performed with virtual hands and textual annotations on the patient avatar. Use cases of the proposed system include remote education of the patient or doctor as well as remote consultations where remote experts could guide local clinicians. A limitation of the proposed system is that it is not intended for use in high-precision tasks such as surgical procedures because of the limited accuracy of matching the avatar and patient body. Future work will include test cases in which clinicians perform real medical procedures. Further evaluation will be performed to obtain the system usability score for different tasks and investigate the agreement between the in-person assessment and the remote examination performed by the proposed system. Latency improvement will also be addressed by the C++ implementation of steps critical for processing speeds.

## Figures and Tables

**Table 1:** Time delay of avatar movement.

Service	Time Delay (ms)
WebRTC P2P	200–300
Grab frame	3–8
Person detection	25–60
Person identification	30–60
3D pose estimation	15–50
WebSocket MOCAP	50
Unity 3D model transformation (running on laptop)	5–15
Unity 3D model transformation (running on HoloLens 2)	10–25

**Table 2:** Performance of the AR and VR client applications.

	AR Build		AR Remote		VR Build	
**Body System**	**No Light (FPS)**	**Light (FPS)**	**No Light (FPS)**	**Light (FPS)**	**No Light (FPS)**	**Light (FPS)**
Visceral system	26	25	60	60	120	120
Muscural system	13	12	60	60	120	120
Cardiovascular system	8	8	60	60	120	120
Nervous system and sence organs	13	13	60	60	120	120
Regions of human body/skin	54	51	60	60	120	120
Skeletal system	28	28	60	60	120	120
Joints	38	36	60	60	120	120
Muscular insertion	26	26	60	60	120	120
Lymphoid organs	50	46	60	60	120	120

## Data Availability

Data are contained within the article.
